# 

**Published:** 1889-07

**Authors:** 


RECEIPTED FOR 1889.—Drs. P. Williams, J. A. Sloan, E. D. Mortor, Martin Sprayer, R. L. Vance, Jas. Geiger, J. J. Poor to May, H H Whataker to June, 90, T S Freeman, to June, '90, I T Young, to '88.

